# Supplementary material for: Deep learning-based grading of ductal carcinoma in situ in breast histopathology images
Source: Lab Invest. 2021 Feb 19;101(4):525–33. doi: 10.1038/s41374-021-00540-6 (PMC7985025; doi:10.1038/s41374-021-00540-6)
Supplement: Supplementary file 1 — Supplementary Methods [file 41374_2021_540_MOESM1_ESM.docx]

**Supplementary Information**

*Input to the deep learning system*

The data acquisition process resulted in whole-slide images (WSI) with outlined ductal carcinoma *in situ* (DCIS) lesions. The DCIS lesions were extracted from the WSI by fitting a rectangular box around the manually annotated lesions. An additional 90µm border was drawn around these boxes in order to include the DCIS lesion as well as the surrounding stroma. These boxes were extracted at magnification level ×10. We cropped random patches of 512 × 512 pixels (about 450 µm × 450 µm) from all DCIS lesion boxes and applied data augmentation during training (not at test time). The data augmentation consisted of translations, rotations, flipping, shearing, zooming and color channel shifts. All augmentations were applied randomly within certain limits. Translations of up to 25% of the image size were applied horizontally and vertically. Images were rotated up to ±90 degrees and flipped horizontally and vertically. The shear intensity, range for zoom and color channel intensity were all changed up to ±20% of their original values. After data augmentation the patches were fed into the network.

*The deep learning system*

The deep learning system used to grade DCIS was based on the DenseNet-121 [1] network architecture. The mini batch size was set to 12 and we balanced the batches to always include 4 patches of each DCIS grade. The networks were trained by minimizing the ordinal categorical cross-entropy loss between the ground truth and the predictions. This loss was calculated as the categorical cross-entropy loss multiplied with the differences in the predicted and ground truth DCIS grade plus 1. We used ImageNet pre-training and the optimization was done with stochastic gradient descent with a learning rate of 1e-4 and momentum of 0.95. Training was stopped when the kappa score on the validation set started decreasing. Hyper-parameters, like the learning rate and the mini batch size, were tuned to optimal performance on the validation set by grid search.

*Output of the deep learning system*

The first output of the system was a DCIS grade (either 1, 2 or 3) for each patch. The second output was a number that predicted how many observers would agree with this grade (either 1, 2 or 3). We did not use this second prediction at test time, but it was used during training to feed the network information about boundary cases.

*Variations across training runs of the deep learning system*

Deep learning methods can have different predictive performance across training runs because of the stochastic nature of gradient descent and the fact that different local minima may be found for each run. To research the variability in DCIS grading for our deep learning system we trained it 5 times with the same hyper-parameter settings. The results in the main manuscript are the results from the first training run. Table 1 shows the mean and standard deviation of the inter-observer agreement over 5 training runs.

**Table 1:** Inter-observer quadratic weighted Cohen’s Kappa for ductal carcinoma in situ (DCIS) grading at the lesion-level and patient-level between three observers and the deep learning system. The results are shown on the test set which contains 1001 lesions from 50 different patients. The Cohen’s Kappa shown is the mean of 5 training runs of the same deep learning algorithm. The standard deviation between the 5 training runs is also shown.

|  | Lesion-level | | Patient-level | |
| --- | --- | --- | --- | --- |
|  | Deep learning system | | Deep learning system | |
|  | mean(*κ*) | SD(*κ*) | mean(*κ*) | SD(*κ*) |
| Observer 1 | 0.76 | 0.03 | 0.69 | *0.06* |
| Observer 2 | 0.50 | 0.02 | 0.70 | 0.04 |
| Observer 3 | 0.41 | 0.01 | 0.74 | 0.03 |

**References**

[1] Huang G, Liu Z, Van Der Maaten L, Weinberger KQ. Densely connected convolutional networks. *In Proc IEEE Comput Soc Conf Comput Vis Pattern Recognit* 2017:4700-4708.
